# Editorial: Explainable artificial intelligence models and methods in finance and healthcare

**DOI:** 10.3389/frai.2022.970246

**Published:** 2022-08-16

**Authors:** Brian S. Caffo, Fabio A. D'Asaro, Artur Garcez, Emanuela Raffinetti

**Affiliations:** ^1^Department of Biostatistics, Johns Hopkins University, Baltimore, MD, United States; ^2^Department of Human Sciences, University of Verona, Verona, Italy; ^3^Department of Computer Science, City University of London, London, United Kingdom; ^4^Department of Economics and Management, University of Pavia, Pavia, Italy

**Keywords:** artificial intelligence, explainability, generalizability, machine learning, parsimony, forecasting

## 1. Foreword

Specialized Artificial Intelligence (AI) is poised to have a profound impact on healthcare and finance. These complex models can be used to forecast, diagnose, explore variation in symptoms or financial metrics, engineer new features and create lower dimensional embeddings. The result is a new field of engineering (Jordan, 2019) that could revolutionize these areas.

First, we should define what we mean by “Artificial Intelligence.” Historically, this term was reserved for the attempt to create computational variations of general intelligence (Pennachin and Goertzel, 2007). While research into general AI continues, much of modern AI research focuses on so-called narrow or specialized AI, which uses AI to solve targeted problems, like object recognition in images or converting speech to text (Pennachin and Goertzel, 2007). As such, specialized AI has no sharp boundaries from machine learning (ML).

Specialized AI is revolutionizing many fields, with radiology as a prime example (Hosny et al., 2018). In radiology, highly skilled and trained medical doctors are required to interpret patient scans for diagnoses or prognoses. The success of specialized AI in this area has been so rapid of late that Dr. Geoffrey Hinton, a Turing award wining AI researcher, famously quipped (while being deliberately controversial) “... we should stop training radiologists” (Alvarado, 2022).

Dr. Hinton's quote envisions a world where much of the decision making in our lives is specialized AI. Healthcare and finance represent two areas where this change is believed to likely to occur rapidly (Aziz and Dowling, 2019; Shaheen, 2021). Algorithms may diagnose our diseases, predict our recovery, predict what are the best treatments, determine whether we get a loan, invest for us and determine the cost of goods and services.

Can we trust the algorithms that will drive our cars and make medical and financial decisions for us? Specifically, trust remains a key required ingredient for large scale AI adoption, in healthcare, finance and elsewhere. Such trust requires the ability to identify how the algorithm is engineering features to create predictions, diagnoses or forecasts, as well as the ability for the algorithm to generalize to novel settings, unrelated to the training, testing and validation datasets used to create the algorithm. In classical statistical models, parsimony, study design and modeling choices were the foundation for obtaining explainability, generalizability and trust in a model. Modern AI engineers have the same amount of control over study design, but often try to automate many of the modeling choices. Achieving parsimony, however, is often not realistic in models with millions of parameters (weights). The goal of explainable AI could therefore be cast as the ability to create variations of parsimony in complex models for the purpose of engendering trust in the algorithm, by detecting the factors which actually impact the phenomenon under evaluation. This latter point should be emphasized as explainable AI, which ideally identifies the features or combinations of features with the greatest impact on the outcome to: motivate future study, build secondary parsimonious models and to build trust in the algorithm by matching feature importance with known or postulated mechanisms.

The practical deployment of AI systems in healthcare and finance has been hampered by this lack of trust. Explainability is seen as a key step in increasing trust and accountability of complex AI systems. In healthcare, patients, caregivers and regulators need to be able to explain and trust AI systems to put them into use. Similarly, financial systems are unlikely to satisfy the stringent regulations of the field without explainability. In each area, there is a need for explainability to create parsimony in complex models. New specialized algorithms and visualization techniques may be needed to provide a window into these complex systems. New metrics are needed too, offering a fair comparison of results, trade-offs and measuring the fidelity of the explanations prior to their production use. More focused, application-oriented work is needed where explainable AI offers the groundwork for trust and accountability in AI.

While the core topic of this special issue is explainability, its core is on trusting algorithmic output. This multifaceted topic at least includes: rigorous validation together with methods for evaluating explainability and generalizability. Each of the manuscripts in the special issue touches one or more of these core issues.

## 2. Review of the special issue manuscripts

In their manuscript “*SHAP and LIME: An Evaluation of Discriminative Power in Credit Risk*,” Gramegna and Giudici explore credit default risk prediction. As they articulate, modern algorithms, including AI, can produce more accurate estimates of the probability of default for a credit applicant. However, this increase in prediction power often comes at the expense of interpretability of the prediction model. They applied an ML model (XGBoost) on an Italian credit risk data set and considered clustering the results of SHAP and LIME. They concluded SHAP slightly outperformed for the discrimination of realized defaults.

Maccarrone et al. focus on a different problem of forecasting the gross domestic product (GDP) using a variety of prediction approaches. They contrasted ML and non-ML approaches for predicting GDP. The KNN approach performed the best of those considered. They considered predicting both a one and multiple financial quarters ahead of the training data. Their contributions to explainability stem from the exploration of different fitting and forecasting validation strategies.

de Lacy et al. introduce Integrated Evolutionary Learning (IEL) which attempts to simultaneously consider model selection and interpretability. Notably, their model considered functional magnetic resonance imaging data (fMRI) and other covariates to predict behavioral and diagnostic outcomes using a measure of daily life function and autism traits separately. They used an evolutionary search algorithm to optimize model hyperparameters *via* a stochastic search and information criterion (BIC) fitness function. Their approach offers several avenues for explainability. First, by contrasting multiple models, including parsimonious regression approaches and a rigorous validation, a general sense of variable and model input is garnered. Secondly, the IEL algorithm yields general variable importance metrics, which can be contrasted within and between algorithms.

Finally, Wan et al. survey a framework for the generalizability of AI algorithms. Generalizability, fairness and AI ethics, as well as explainability in AI all go hand in hand. The authors create a Rosetta Stone between traditional epidemiological and biostatistical clinical research concepts and modern AI dataset shift. Their work focuses on clinical prediction algorithms, where generalizability is a key concept.

## 3. Discussion

Explainability and trust represent only one of several key bottlenecks to the widespread adoptions of AI in healthcare and finance. Panch et al. (2019) also mention the difficulties in incorporating AI into existing complex systems and training data availability as two important hurdles. Wan et al. discuss dataset shift; however, dataset shift still presumes data availability. Small datasets or lack of data prevents training the complex models required for AI. Often, the data exists, but is not available to build models. This is commonplace in healthcare, where data exists to support patient care. Data organization and privacy considerations often prevent creating the large, processed, high quality datasets needed to train AI.

Incorporating AI into existing complex systems was the other concern raised in Panch et al. (2019). Whether approving loans or diagnosing diseases, swapping out narrow tasks, even with AI that is narrowly superior to the human agents, remains difficult if the surrounding tasks are interconnected, requiring interaction with the agent. This is almost always the case.

We conclude with a discussion of AI ethics. Explainability and trust go hand in hand with ethical AI development. Being able to explain how an algorithms is obtaining predictions allows one to evaluate biases. Sand et al. (2022) outline this, but also include transparency, accountability, privacy and other considerations. This, once again, highlights that explainability remains only one of several related gaps that need to be addressed to have wide scale AI implementation in healthcare and finance.

## Author contributions

All authors listed have made a substantial, direct, and intellectual contribution to the work and approved it for publication.
