# Corrigendum: Editorial: Explainable artificial intelligence models and methods in finance and healthcare

**DOI:** 10.3389/frai.2023.1157762

**Published:** 2023-02-20

**Authors:** Brian S. Caffo, Fabio A. D'Asaro, Artur Garcez, Emanuela Raffinetti

**Affiliations:** ^1^Department of Biostatistics, Johns Hopkins University, Baltimore, MD, United States; ^2^Department of Human Sciences, University of Verona, Verona, Italy; ^3^Department of Computer Science, City University of London, London, United Kingdom; ^4^Department of Economics and Management, University of Pavia, Pavia, Italy

**Keywords:** artificial intelligence, explainability, generalizability, machine learning, parsimony, forecasting

In the published article, there was an error in the Acknowledgments statement. The mistake was related to the part referring to the ER acknowledgments, and more precisely in the Grant Agreement No. of the European xAIM (eXplainable Artificial Intelligence in healthcare Management), which was incorrectly written as INEA/CEF/ICT/A2020/2265375. The correct Acknowledgments statement appears below:

